# Associations between maternal stress during pregnancy and offspring internalizing and externalizing problems in childhood

**DOI:** 10.1186/1752-4458-8-44

**Published:** 2014-11-18

**Authors:** Subin Park, Bung-Nyun Kim, Jae-Won Kim, Min-Sup Shin, Hee Jeong Yoo, Jin Lee, Soo-Churl Cho

**Affiliations:** Department of Psychiatry, Seoul National Hospital, Seoul, Republic of Korea; Department of Psychiatry and Behavioral Medicine, Seoul National University College of Medicine, Seoul, Republic of Korea; Division of Child and Adolescent Psychiatry, Department of Psychiatry, Seoul National University Hospital, 101 Daehak-No, Chongno-Gu, Seoul, Korea; Department of Psychiatry, Seoul National University Bungdang Hospital, Seongnam, Republic of Korea

**Keywords:** Internalizing problems, Externalizing problem, Perinatal risk factors

## Abstract

**Background:**

Maternal psychological health during pregnancy has been associated with offspring psychopathology. However, it is uncertain whether these associations are mediated by the postpartum depression and related child-rearing factors. Therefore, we examined the associations between prenatal and postnatal factors and internalizing and externalizing behavioral problems in childhood, focusing on maternal psychological health in school-aged children in Korea.

**Findings:**

The current study included 1,003 children (580 boys, 423 girls, mean age 9.05 ± 0.70 years, age range 8–11 years) recruited from schools in five Korean cities. Children’s internalizing and externalizing problems were assessed by the Child Behavior Checklist (CBCL). The parents of the children completed structured questionnaires on perinatal factors. Among 1,003 children, 44 had internalizing problems (IP) and 30 had externalizing problems (EP). When comparing children with IP (n = 44) and without IP (n = 959), severe maternal stress during pregnancy (OR3.36, 95% CI 1.80-6.25) and postpartum depression (OR3.19, 95% CI 1.36-7.53) showed a significant association with the IP. When comparing children with EP (n = 30) and without EP (n = 973), low family income (OR2.19, 95% CI 1.05-4.56), unwanted pregnancy (OR2.76, 95% CI 1.28-5.95) and severe maternal stress during pregnancy (OR2.69, 95% CI 1.29-5.61) with the EP. Only maternal stress during pregnancy was significantly associated with the IP after controlling for postpartum depression and with the EP after controlling for family income and unwanted pregnancy.

**Conclusion:**

This study suggests the importance of maternal psychological health during perinatal period on children’s mental health. Further prospective studies in a larger sample are required to confirm our findings.

## Background

Fetal growth is a sensitive period in which maternal and environmental insults, including those due to both maternal physical and psychological health factors such as depression and severe stress during pregnancy, can alter fetal development and have a lasting impact on the offspring’s neurological and behavioral development [1, 2]. Both environmental and inherited influences may explain the association between prenatal stress or psychopathology and offspring neurobehavioral development [3, 4]. Mothers who experience prenatal depression or anxiety are more likely to be depressed and anxious in the months and years following pregnancy [5], and postpartum depression has been associated with parenting stress, impacting attachment and child development [6, 7]. Severe stress during pregnancy and related postpartum depression may also be associated with risk factors in early childhood such as postpartum parenting stress and change in primary care taker [8]. Therefore, these factors should be comprehensively considered when examining the impact of maternal psychological health in the perinatal period on offspring psychopathology.

Previous studies have shown that maternal stress experienced during pregnancy is strongly associated with offspring behavioral and emotional problems [9–13]. Perceived stress during pregnancy was associated with an increased risk of offspring pediatric disorders including mental disorders [9], temperamental variation of young infants [10], and more behavioral problems in 2-year-olds [11]. Prenatal maternal depressive, anxious, and stress symptoms was related to offspring internalizing and externalizing behaviors and depressive symptoms [12]. Children with attention-deficit hyperactivity disorder whose mothers were exposed to moderate and severe stress during pregnancy tended to develop more severe symptoms than children with attention-deficit hyperactivity disorder whose mothers were not exposed to prenatal stress [13]. However, it is uncertain whether these associations are mediated by the postpartum depression or the related child-rearing factors. In other words, it remains unknown whether the association between maternal stress during pregnancy and offspring behavioral and emotional problems is significant even after controlling for postnatal maternal and child-rearing factors.

In this investigation, we attempted to further clarify the complex relationships between prenatal and postnatal maternal psychological health, other perinatal risk factors, and offspring internalizing and externalizing behavioral problems in childhood. We hypothesized that severe maternal stress experienced during pregnancy is independently associated with childhood internalizing and externalizing behavioral problems, even after controlling for perinatal risk factors including postpartum depression.

## Methods

### Participants and procedure

Participants were recruited from five different administrative regions of Korea - Seoul, Seongnam, Incheon, Ulsan, and Yeoncheon. We selected two to three schools from each region, for a total of thirteen schools, and sent letters to the parents of third and fourth grade children inviting them to participate in our study. We gave the parents and children detailed information about the study and then obtained written informed consent before any child entered the study. Of the initially recruited 1,089 children, 86 study subjects were excluded because their responses were incomplete, leaving a total of 1,003 subjects (92.1%; 580 boys, 423 girls, mean age 9.05 ± 0.70 years) in the analysis. Among 1,003 children included in the study, 44 (25 boys, 19 girls) had internalizing problems and 30 (19 boys, 11 girls) had externalizing problems. The study participants’ geographic distribution was as follows: 413 (41.2%) from urban districts, 400 (39.9%) from industrial cities, and 190 (18.9%) from the rural district. The study protocol was approved by the institutional review board of the Seoul National University Hospital.

Trained laypersons conducted face-to-face interviews of the mothers at each participant’s school. Mothers were asked about structured questionnaire on perinatal risk factors**.** It takes about 30 minutes for completion of the questionnaires. Mothers were asked about their age at pregnancy and whether they had wanted pregnancy (wanted or unwanted), whether they had regular prenatal checkups (regular, irregular, or no prenatal checkups), and whether they had consumed alcohol during pregnancy (yes or no; if yes, 0–1 drink per day, 2–3 drinks per day, 4–5 drinks per day, and 6 more drinks per day). Because 97% of women who had consumed alcohol during pregnancy reported drinking between 0 and 1 drink per day, alcohol consumption was analyzed as a dichotomous variable (yes or no). Mothers were also asked about whether they had experienced any personal or social situations that caused severe physiological and psychological stress during pregnancy (e.g., conflicts with family members, financial difficulty, illness or death of relatives, pregnancy complication) (yes or no; if yes, specify the stressor). Mothers were asked whether they had experienced persistent depressive symptom (e.g., depressed mood or loss of interest, changes in appetite or sleep, psychomotor retardation or agitation, guilty feeling, suicidal idea) for at least 1 month in the first year postpartum (yes or no). The severity of postpartum depression was assessed using a 5-point Likert-type scale (1, very mild to 5, very severe) and categorized as none, very mild to mild (1–2), and moderate to very severe (3–5). The primary caretaker during the first year was assessed (response options were mother, father, grandparent, babysitter, nursery, or other) and categorized as either mother or any caretaker other than mother. Number of changes in the primary caretaker from birth to 3 years of age was assessed and categorized as either none or one plus.

Mothers completed the Korean version of the Child Behavior Checklist (K-CBCL) [14, 15] to evaluate internalizing and externalizing symptoms of children. The CBCL produces three broadband scores (i.e., internalizing, externalizing, and total problem scores) that can be compared to norms and clinical cutoffs for groups basedon age and sex. We considered T-scores of 63 and above to signify clinically significant symptoms based on previous validation studies. In the study by Oh et al. [15], 70 T was considered to be an appropriate cut-off point in a clinical sample, but 63 T was recommended as the most effective cut-off point to screen problem behavior in community sample.

Childrenwith internalizing problem (IP) scores of 63 T and above were placed in the IP group and children with the scores below 63 T were placed in the control group without IP. Children with externalizing problem (EP) scores of 63 T and above were placed in the EP group and children with the scores below 63 T were placed in the control group without EP.

### Statistical analysis

Group differences in demographic and perinatal variables were evaluated using binary logistic regression tests. Two sets of dichotomized outcomes were defined as the IP group versus controls without IP, and the EP group versus controls without EP. The predictive variables were demographic (child’s age, sex, region, family income, parental education levels), prenatal (maternal age at pregnancy, unwanted pregnancy, no regular prenatal check-ups, maternal alcohol intake, severe stress during pregnancy), and child-rearing factors (change in primary care taker, primary care taker other than mother, postpartum depression).

First, individual variables were independently included in the logistic model one by one with no other covariates. Next, the variables significantly different between group at the level of p <0.05 in the univariate analysis were concurrently entered into the model, and for each individual variable, odd ratios adjusted for other variables-all variables concurrently entered in the multivariate model except for itself- were calculated.

To overcome the disproportionate ratio between the IP or EP group and controls, we selected 10% of the sample from control group using the ‘Selected Cases’ in SPSS, and compared perinatal variables between a random sample from control group without IP (N = 96) and the IP group (N = 44) and between a random sample from control group without EP (N = 97) and the EP group (N = 30) using binary logistic regression tests.

All statistical analyses were performed using SPSS (version 21.0), and statistical significance was defined at the alpha level <0.05.

## Results

First, we compared demographic characteristics of children with the IP or the EP compared to controls to identify possible confounding variables in the main analysis (comparison of perinatal risk factors between the IP or EP group and the control group). The EP group were more likely to have low family income than control group without EP (OR = 2.19, 95% CI = 1.05-4.56). Other demographic variables were not significantly different between groups (Table 1).

**Table 1:** **Characteristics of children with the Internalizing Problems (IP) or with the Externalizing Problems (EP) compared to controls**

	Controls without IP (N = 959)	IP (N = 44)		Controls vs. IP		Controls without EP (N = 973)	EP (N = 30)		Controls vs. EP	
			UOR	d (95% CI)	p			UOR	d (95% CI)	p
Age, mean (SD)	9.04 (0.70)	9.20 (0.59)	-1.49^a^		0.137^b^		9.03 (0.72)	0.14^a^		0.885^b^
Male (n(%))	560 (57.9)	25 (56.8)	0.96 (0.52-1.76)	-0.10 (-0.25-0.05)	0.890	561 (57.7)	19 (63.3)	1.27 (0.60-2.70)	0.06 (-0.13-0.25)	0.536
Region (n(%))										
Urban	404 (41.8)	17 (38.6)	ref			413 (42.1)	8 (26.7)	ref		
Industrial	381 (39.4)	19 (43.2)	1.17 (0.64-2.15)	0.04 (-0.10-0.18)	0.616	384 (39.1)	16 (53.3)	1.78 (0.86-3.68)	0.14 (-0.04-0.32)	0.122
Rural	182 (18.8)	8 (18.2)	1.04 (0.48-2.28)	0.01 (-0.18-0.20)	0.915	184 (18.8)	6 (20.0)	0.92 (0.37-2.29)	-0.02 (-0.24-0.20)	0.864
Family income (n(%))										
Middle or upper	601 (62.2)	26 (59.1)	ref			596 (62.6)	13 (43.4)	ref		
Lower	366 (37.8)	18 (40.9)	1.14 (0.62-2.10)	0.03 (-0.12-0.18)	0.683	356 (37.4)	17 (56.7)	2.19 (1.05-4.56)	0.19 (0.01-0.37)	0.036^*^
Paternal education (n(%))										
High school degree or lower	514 (53.2)	28 (64.3)	1.59 (0.83-3.02)	0.11 (-0.04-0.26)	0.161	523 (53.3)	20 (65.5)	1.67 (0.77-3.62)	0.12 (-0.07-0.31)	0.198
College degree or higher	453 (46.8)	16 (35.7)	ref			458 (46.7)	10 (34.5)	ref		
Maternal education (n(%))										
High school degree or lower	647 (66.9)	31 (71.4)	1.23 (0.62-2.44)	0.05 (-0.12-0.22)	0.550	656 (66.9)	23 (75.9)	1.55 (0.66-3.68)	0.10 (-0.11-0.31)	0.316
College degree or higher	320 (33.1)	13 (28.6)	ref			325 (33.1)	7 (24.1)	ref		

^a^t-statistics; ^b^result of independent t-test, others are the results of binary logistic regression analyses; UOR, unadjusted odd ratio; ref, reference group; ^*^p <0.05.

Next, we conducted univariate logistic regression analyses including prenatal and child-rearing factors as a predictive variable and the presence of IP or EP as an outcome variable. Severe maternal stress during pregnancy (OR = 3.36, 95% CI = 1.80-6.25) and postpartum depression (OR = 3.19, 95% CI = 1.36-7.53) showed a significant association with the internalizing problems and unwanted pregnancy (OR = 2.76, 95% CI = 1.28-5.95) and severe maternal stress during pregnancy (OR = 2.69, 95% CI = 1.29-5.61) with the externalizing problems (Table 2). These significant associations remained in the comparison between a 10% random sample from control group and the IP or EP group (Table 3).

**Table 2:** **Perinatal factors of children with the Internalizing Problems (IP) or with the Externalizing Problems (EP) compared to controls**

	Controls without IP (N = 959)	IP (N = 44)	Controls vs. IP			Controls without EP (N = 973)	EP (N = 30)	Controls vs. EP		
	%	%	UOR	d (95% CI)	p	%	%	UOR	d (95% CI)	p
Maternal age at pregnancy										
Below 30	67.8	61.9	ref			68.0	55.2	ref		
30 years or above	32.2	38.1	1.30 (0.69-2.46)	0.06 (-0.09-0.21)	0.423	32.0	44.8	1.72 (0.82-3.63)	0.13 (-0.05-0.31)	0.152
Unwanted pregnancy	28.3	29.0	1.68 (0.85-3.09)	0.12 (-0.03-0.27)	0.144	28.1	53.3	2.76 (1.28-5.95)	0.24 (0.06-0.42)	0.010^*^
No regular prenatal check-ups	10.8	9.3	0.84 (0.30-2.41)	-0.04 (-0.21-0.29)	0.750	10.8	10.0	0.95 (0.28-3.21)	-0.01 (-0.31-0.29)	0.939
Maternal alcohol intake	3.9	6.8	1.79 (0.53-6.04)	0.14 (-0.16-0.44)	0.349	3.9	10.0	2.76 (0.80-9.49)	0.24 (-0.78-1.26)	0.108
Severe stress during pregnancy	32.1	61.4	3.36 (1.80-6.25))	0.29 (0.14-0.44)	<0.001^**^	32.7	56.7	2.69 (1.29-5.61)	0.24 (0.06-0.42)	0.008^**^
Change in primary care taker	14.6	18.6	1.34 (0.61-2.95)	0.07 (-0.12-0.26)	0.467	14.8	13.3	0.89 (0.31-2.58)	-0.03 (-0.29-0.23)	0.815
Primary care taker other than mother	21.0	20.5	0.97 (0.46-2.06)	-0.01 (-0.20-0.18)	0.932	20.8	26.7	1.39 (0.61-3.16)	0.08 (-0.12-0.28)	0.438
Postpartum depression										
None	73.4	52.6	ref			72.5	68.0	ref		
Very mild to mild	17.4	26.3	2.10 (0.96-4.60)	0.18 (-0.01-0.37)	0.063	17.7	24.0	1.45 (0.56-3.74)	0.09 (-0.15-0.23)	0.444
Moderate to severe	9.2	21.1	3.19 (1.36-7.53)	0.28 (0.07-0.49)	0.008^**^	9.8	8.0	0.87 (0.20-3.84)	-0.03 (-0.39-0.33)	0.853

Results of binary logistic regression analyses; UOR, unadjusted odd ratio; ref, reference group; ^*^p <0.05.^**^p <0.01.

**Table 3:** **Perinatal factors of children with the Internalizing Problems (IP) or with the Externalizing Problems (EP) compared to a random sample from controls**

	Controls without IP (N = 96)	IP (N = 44)	Controls vs. IP			Controls without EP (N = 97)	EP (N = 30)	Controls vs. EP		
	%	%	UOR	d (95% CI)	p	%	%	UOR	d (95% CI)	p
Maternal age at pregnancy										
Below 30	68.1	61.9	ref			68.0	55.2	ref		
30 years or above	31.9	38.1	1.31 (0.62-2.80)	0.07 (-0.12-0.25)	0.482	32.0	44.8	1.45 (0.61-3.45)	0.09 (-0.12-0.30)	0.399
Unwanted pregnancy	25.0	39.0	1.92 (0.86-4.27)	0.16 (-1.80-2.12)	0.109	28.1	53.3	3.55 (1.40-8.99)	0.30 (0.08-0.53)	0.008^**^
No regular prenatal check-ups	14.0	9.3	0.63 (0.19-2.06)	-0.11 (-0.40-0.18)	0.446	10.8	10.0	1.75 (0.39-7.85)	0.13 (-0.23-0.50)	0.463
Maternal alcohol intake	4.0	6.8	1.74 (0.37-8.12)	0.13 (-0.25-0.51)	0.482	3.9	10.0	2.22 (0.47-10.57)	0.19 (-0.19-0.57)	0.316
Severe stress during pregnancy	37.6	61.4	2.63 (1.26-5.50))	0.23 (0.05-0.41)	0.010^*^	32.7	56.7	2.35 (1.01-5.50)	0.21 (-0.00-0.41)	0.048^*^
Change in primary care taker	11.1	18.6	1.83 (0.67-5.03)	0.15 (-0.10-0.39)	0.242	14.8	13.3	0.54 (0.17-1.75)	-0.15 (-0.43-0.14)	0.302
Primary care taker other than mother	21.2	20.5	0.96 (0.40-2.30)	-0.01 (-0.22-0.20)	0.918	20.8	26.7	1.55 (0.58-4.10)	0.11 (-0.14-0.35)	0.382
Postpartum depression										
None	737	52.6	ref			72.5	68.0	ref		
Very mild to mild	16.2	26.3	2.28 (0.90-5.79)	0.20 (-0.03-0.43)	0.083	17.7	24.0	1.25 (0.41-3.81)	0.05 (-0.22-0.33)	0.696
Moderate to severe	10.1	21.1	2.92 (1.02-8.37)	0.26 (-0.00-0.52)	0.046^*^	9.8	8.0	0.90 (0.17-4.91)	0.03 (-0.39-0.44)	0.905

Results of binary logistic regression analyses; UOR, unadjusted odd ratio; ref, reference group; ^*^p <0.05.^**^p <0.01.

Then, we conducted multiple logistic regression analyses including severe maternal stress during pregnancy and postpartum depression (the variables different between the IP group and controls in the univariate analysis) as a predictive variable and the presence of IP as an outcome variable. In this model, severe maternal stress during pregnancy (adjusted OR [AOR] =3.09, 95% CI = 1.51-6.31, p = 0.002) was significantly associated with IP, but postpartum depression was not (AOR = 1.72, 95% CI = 0.77-3.81, p = 0.185 for very mild to mild depression vs. no depression and AOR = 2.07, 95% CI = 0.85-5.06, p = 0.110 for moderate to very severe depression vs. no depression). Finally, we conducted multiple logistic regression analyses including family income, unwanted pregnancy, and severe maternal stress during pregnancy (the variables different between the EP group and controls in the univariate analysis) as a predictive variable and the presence of EP as an outcome variable.In this model, severe maternal stress during pregnancy (AOR = 2.41, 95% CI = 1.08-5.35, p = 0.031) was significantly associated with EP, but unwanted pregnancy (AOR = 2.15, 95% CI = 0.97-4.73, p = 0.058) and lower family income (AOR = 1.83, 95% CI = 0.84-4.02, p = 0.130) were not significantly associated with EP. Taken together, severe maternal stress during pregnancy was a significant risk factor associated with both IP and EP even after controlling for other risk factors.

Finally, we evaluated differences in perinatal variables between a random sample from control group and IP or EP group using binary logistic regression tests.

Conducted univariate logistic regression analyses including prenatal and child-rearing factors as a predictive variable and the presence of IP or EP as an outcome variable. Severe maternal stress during pregnancy (OR = 3.36, 95% CI = 1.80-6.25) and postpartum depression (OR = 3.19, 95% CI = 1.36-7.53) showed a significant association with the internalizing problems and unwanted pregnancy (OR = 2.76, 95% CI = 1.28-5.95) and severe maternal stress during pregnancy (OR = 2.69, 95% CI = 1.29-5.61) with the externalizing problems (Table 2).

## Discussion

In this study, we found that severe maternal stress experienced during pregnancy was highly associated with both IP and EP of school-aged children. In addition, post-partum depression was associated with IP and low family income and unwanted pregnancy were associated with EP, but these associations did not remain after controlling for maternal stress during pregnancy.

Consistent with our results, previous studies have shown that maternal stress experienced during pregnancy might be strongly related to offspring psychopathology [9–13]. In a national cohort study conducted in Denmark, maternal stress during pregnancy was highly associated with pediatric mental disorders (age 0–2.5 years: OR =2.03) as well as physical diseases after controlling for maternal stress after pregnancy [9]. Recently, using the data of Mater University Study of Pregnancy (MUSP), an Australian-based, prebirth cohort study, Betts et al. [12] reported that maternal depression, anxiety, and stress symptoms in early pregnancy uniquely increased the risk of internalizing behavior problems in adolescence. The authors also reported the lack of an association between postnatal maternal depressive, anxious, and stress symptoms with offspring behavior problems, suggesting that prenatal factorsare more strongly related to offspring internalizing problems than postnatal factors. With regard to externalizing problems, Gutteling et al. [10] examined 103 healthy toddlers in a prospective study and found that perceived stress during pregnancy was a predictor of lower levels of restless/disruptive temperament (OR = 0.77), more total behavioral problems (OR = 1.17), and more externalizing behavioral problems (OR = 1.12) in 2-year-olds.In a prospective study of nulliparous Scandinavian women, exposure to prenatal stress was related to attention-deficit hyperactivity disorder, the most common externalizing behavioral disorder, in their offspring at 7 years [16].

Although highly correlated with psychiatric symptoms, self-perceived stress is considered separate from depression and anxiety [1] and is often conceptualized as a manifestation of the subjective physical and mental state in response to a broad range of day-to-day problems and life events [9, 17]. In the present study, the prenatal stressors reported by mothers included conflicts with family members, loneliness, parenting stress associated with the older child, financial difficulty, illness or death of relatives, and physical problem such as emesis or pregnancy complication. Many of these factors can be preventable through family education and public support such as social services for pregnant women and low-income assistance.

The limitations of this study include a lack of reliability and validity testing for the single-item surveys on maternal stress during pregnancy and postpartum depression. Moreover, the assessment of offspring behavior was relied on maternal reports, which are likely to be biased by mother’s own psychological health. Another limitation is that, because the data on prenatal and child-rearing factors were based on the recollection of the mothers of the children, the respondents’ reports may be characterized by inaccuracies. In addition, we did not get information on psychiatric family history other than postpartum depression, which could confound the results. Finally, the small sample size of the IP and the EP group did not provide sufficient statistical power to detect modest differences. Therefore, negative findings must be interpreted with caution. Therefore, further prospective studies in a larger sample are required to confirm or refute the perinatal risk factors found in this retrospective study.

In conclusion, this study suggests the importance of maternal psychological health during pregnancy on children’s mental health. In particular, severe maternal stress during pregnancy was the most significant risk factor for both the IP and EP of children independent of other perinatal risk factors such as postpartum depression or unwanted pregnancy. A policy of stress prevention and management for pregnant women should be established to prevent offspring internalizing and externalizing problems.
